# Targeted, actionable and fair: Reviewer reports as feedback and its effect on ECR career choices

**DOI:** 10.1093/reseval/rvad034

**Published:** 2023-11-02

**Authors:** Gemma Elizabeth Derrick, Alessandra Zimmermann, Helen Greaves, Jonathan Best, Richard Klavans

**Affiliations:** School of Education, Centre for Higher Education Transformations (CHET), University of Bristol, Bristol, UK; Proposal Analytics, SciTech Strategies, Inc. Wayne, PA, USA; School of Education, Centre for Higher Education Transformations (CHET), University of Bristol, Bristol, UK; The Wellcome Trust, 215 Euston Road, London NW1 2BE, UK; Proposal Analytics, SciTech Strategies, Inc. Wayne, PA, USA

**Keywords:** peer review, qualitative, research careers, early career researchers, research behaviour

## Abstract

Previous studies of the use of peer review for the allocation of competitive funding agencies have concentrated on questions of efficiency and how to make the ‘best’ decision, by ensuring that successful applicants are also the more productive or visible in the long term. This paper examines the components of feedback received from an unsuccessful grant application, is associated with motivating applicants career decisions to persist (reapply for funding at T_1_), or to switch (not to reapply, or else leave academia). This study combined data from interviews with unsuccessful ECR applicants (*n* = 19) to The Wellcome Trust 2009–19, and manual coding of reviewer comments received by applicants (*n* = 81). All applicants received feedback on their application at T_0_ with a large proportion of unsuccessful applicants reapplying for funding at T_1_. Here, peer-review-comments-as-feedback sends signals to applicants to encourage them to *persist* (continue) or *switch* (not continue) even when the initial application has failed. Feedback associated by unsuccessful applicants as motivating their decision to resubmit had three characteristics: actionable; targeted; and fair. The results lead to identification of standards of feedback for funding agencies and peer-reviewers to promote when providing reviewer feedback to applicants as part of their peer review process. The provision of quality reviewer-reports-as-feedback to applicants, ensures that peer review acts as a participatory research governance tool focused on supporting the development of individuals and their future research plans.

## 1. Introduction

Peer review, despite claims of inefficiency and bias, remains the primary method of decision-making within academia. Despite this prominence in academic decision-making globally, its reputation is jeopardized by claims of bias, time-inefficiency, low cost-effectiveness and a disproportionately high rate of false positive outcomes. Indeed, these claims have led to a number of alternatives proposed, including lottery systems to dictate capitalizing on claims of luck in achieving funding, and streamlined application systems that require less information from applicants but that result on less information for reviewers to base decisions.

Underpinning claims of inefficiency is the realization that peer review, as a process, is characterized by low success rates (Heyard et al. 2022), resulting in the vast majority of researchers being unsuccessful. Despite this silent majority, experiences, career choices and opinions of unsuccessful candidates are largely overlooked in current studies of peer review. In academia, a large part of funding success and failure is dependent on the peer review process and its ability to filter and select applicants towards a binary successful or unsuccessful outcome. In the early phases of academic careers, a period that is characterized by increased competition, precarity and vulnerability for individuals (Browning, Thompson and Dawson 2016; Hamann 2019), achieving initial success in gaining funding is vital and associated with a higher probability of future success of funding, as well as gaining other measures of academic esteem (Merton and Merton 1968; Zuckerman 1977).

For grant decision making, peer review requires researchers, or peers, to read, assess and recommend action on a fellow researcher’s piece of work. Written reports from peer-reviewers are then used by a panel and funding body to assist decision-making as to which research, or researchers to fund. For some funding agencies, such as The Wellcome Trust (Welcome), external reviewer reports are forwarded to the applicants at the conclusion of the decision-making process to indicate reasons for the funding outcome. This research investigates how peer review processes, through the provision of feedback in the form of the peer-review reports, foster the unique career and developmental needs of Early Career Researcher (ECRs).

Specifically, this article aims to address the following research questions;

1. How are the choices of applicants to reapply for funding after receiving an initial unsuccessful outcome (T_0_) based on the feedback received from reviewers?
2. What are the characteristics of reviewer feedback that applicants report influence these choices?

This article focuses on the experiences of unsuccessful candidates and their recollections of how the reviewer reports as feedback influenced their next career decisions. It focuses on the effect of feedback received at T_0_ (initial Wellcome Trust application) when the applicant received an unsuccessful outcome, to inform the decision to reapply for funding, or not, at T_1_ (reapplication).

## 2. Literature review

### 2.1 Early career researchers (ECRs) and funding agencies

Research funding bodies hold a unique custodial role for early career researchers (ECRs). Currently, funding agencies provide many of the fellowship or starting-grant opportunities that can be used to enter, or facilitate a successful academic career (Browning, Thompson and Dawson 2016; McAlpine 2016; McAlpine et al. 2016). They consider the needs of ECRs by acting as signatories on guidelines such as the ECR Concordat in the UK, provide mentoring programmes within successful fellowships, and/or building in mobility clauses, but they have done little to adapt peer review processes to the unique needs of ECRs. Although the definition of an Early Career Researcher (ECR) differs depending on the research and/or funding organization, it is generally acknowledged that this group of young researchers face unique challenges, and vulnerabilities. Specifically, the ECR part of an academic career is characterized by the meta-morphosis from the ‘dependent apprentice’ stage to the ‘colleague-independent’ stage (Laudel and Gläser 2008), and yet, the current competitiveness within academic careers generally, and also in relation to achieving funding at the ECR-level, means that it is becoming more difficult for ECRs to obtain this necessary independence. Indeed, at the Wellcome Trust alone, the award rate for ECR Fellowships has declined from 19% to 12% in the last 5 years, fuelled by a parallel increase in the number of applications received for consideration (37% increase in applicants in the last 5 years), as well as a 3% decrease in the number of awards given. And yet, the burden of early success is felt disproportionately on ECRs, as obtaining a grant/fellowship at the ECR-stage is an important marker of esteem and independence and has been shown to be associated with a greater likelihood of academic ‘successes’ at the later stages of a career (van den Besselaar and Sandström 2015; Bol, de Vaan and van de Rijt 2018; Győrffy, Herman and Szabó 2020).

At the early stages of an academic career, ECRs must simultaneously and progressively: learn to successfully negotiate between the different funding streams on offer (both nationally and internationally); sustain their levels of motivation despite low funding success rates; build a competitive research profile; and, make steps to secure their next contract (McAlpine 2020). Indeed, many of the challenges facing ECRs stem from the precarious nature of academic contracts teamed with a hypercompetitive academic system (Browning, Thompson and Dawson 2016). In addition, access to formal academic mentoring, the ability to travel internationally, network and the associated opportunity to learn from failure is less guaranteed for ECRs when compared to their mid- or late- career colleagues. There is also a high degree of career and financial risk associated with the time taken to apply for funding (von Hippel and von Hippel 2015; Murray et al. 2016), as well as risk associated with possible negative outcomes which are more pronounced for ECRs than for other, more-experienced researchers.

The peer review process, as it is operationalized by funding agencies, plays a large role in dictating future success (Merton and Merton 1968; Bol, de Vaan and van de Rijt 2018; Wang, Jones and Wang 2019). Oettl (2012) called the binary association of success in academic careers a ‘performance taxonomy’ where researchers are classified as either ‘star’ versus ‘non-star’ depending on their ability to attract sufficient academic capital (including funding success) at an early stage. This classification system is also predominant in current research into peer review systems which focuses on outcomes (short and long-term) over social behaviour (Oettl 2012) and other forms of research contribution (McAlpine et al. 2018, McAlpine 2020).

Although not always explicitly acknowledged, funding agencies act as one important actor in promoting the health of the academic research system and research culture more generally. Along with this responsibility, there is a potential for peer review as a process to build on its dominant decision-making function, to also foster a system of reviews-as-feedback to the benefit of all applicants (successful and unsuccessful). Extending this function negates arguments that advocate the removal of peer review entirely. Therefore, there is room to develop a peer review system that balances critique, integrity and transparency of an agency’s peer review processes, with the custodial role of funding agencies to support a strong future academic workforce. Feedback provided to applicants that clearly outlines the reasons for the decision made, as well as constructs a future for the research and researcher, plays a role in this transformation. In the following section, we use literature on effective feedback provision in teaching and learning, to theorize its role within a system of peer review for ECRs.

### 2.2 Signals to persist offered through peer reviewer reports

ECRs are particularly vulnerable (McKenzie 2021) to outcomes dictated by a customary, summative peer review process which dictates the prospects of individual careers even before they can begin. Machine learning approaches link the acquiring of these notions of excellence and indicators of esteem early on in a career with justification for basing future public-funding allocations one these areas of researchers more guaranteed of future success. Although this approach seems an attractive option to streamline an otherwise burdensome peer review evaluation process, its dependence on past success is unlikely to be relevant for ECRs who have had little opportunity to accumulate these indicators of esteem. It also leaves little recognition of the ability of individuals to alter their research career directions, choices or fate as they (as individuals), society or research learn and adapt over time.

Current studies assume that individual researchers and research proposals tap in, and out of the peer review system in pursuit of a binary ‘successful/Yes’ or ‘unsuccessful/No’ decision, where the primary aim of the system is to assess its ‘excellence’. Here, successful applicants are awarded funding, but that their engagement with the peer review system, and hence the peer review system’s responsibility to applicants is limited to obtaining this ‘Stop/Go’ outcome. In this model, successful applicant (‘Yes/Go’) applicants are gifted with funding. In reality, considering the time necessary to prepare grant applications (von Hippel and von Hippel 2015) and the level of professional and emotion investment in its preparation, assessment and development means that it is unlikely that an unsuccessful (Stop/No) decision would result in the research idea being halted entirely. Indeed, a study by Wang, Jones and Wang (2019) investigated the long-term successes of applicants who had missed out on funding (near-miss) versus those who had a ‘near-win’ or just received funding. The results showed that despite an early ‘near miss’, applicants then went on to produce higher impact publications up to 10 years post (negative) grant outcome, than those who had been successful in the same funding round (Wang, Jones and Wang 2019). Whereas the authors use these results to illustrate the ‘randomness’ of peer review decision making for funding allocation, and use this as a reason to promote a lottery-style system of allocation, its findings also show how, if properly harnessed, signals sent through feedback on failed applications may have in encouraging perseverance in individuals, and the further development of their research interests and agenda. Contrary to the conclusions drawn by the Wang, Jones and Wang (2019) article, this study queries what role the signals sent through peer review may play in cementing and encouraging the future success of these ‘near misses’. This signal would explain why some applicants were observed to be more successful in the long run, despite their early ‘near miss’ in attracting funding.

This current study proposes that good feedback provided at the early stages of a research career can be used to further develop research agendas and careers. This ‘signal’ is investigated in the mode and manner of reviewer feedback provided to unsuccessful applicants at the time of receiving their competitive funding outcome.

### 2.3 Components and delivery of good feedback for future learning

Feedback is defined as a process where learners make sense of information, and then use it to enhance their work and strategies (Boud and Molloy 2013b; Ajjawi and Boud 2017; Carless and Boud 2018). Beyond the PhD, the provision of feedback on submitted work—especially for grant and promotion applications—primarily provides judgements and outcomes only, rather than providing information to foster the future development of ideas or applicants. Instead, applicants (and applications) receive a combination of binary outcomes: they win, or lose; they are funded, or they are not; they are accepted or rejected. Outcomes are predominantly explained through political or organizational rationales for the decisions, rather than providing individuals with actionable points to foster future ambitions. More importantly, the current system only fulfils one of the aspects of the feedback definition but fails in the other, arguably more important half of the definition, which is that learners must then ‘use’ the information to enhance their work and future strategies.

Currently, the vast majority of funding agencies see reviewer reports as akin to feedback as a product (Nicol 2010; Price et al. 2013) where its components are concentrated with regulations such as content, length, style and legibility (Orsmond and Merry 2011) through the provision of reviewer templates or clearly articulating evaluation criteria. In this way, feedback acts to reinforce criteria and acts to defend the credibility to the outcome and decision-making process. Alternatively, feedback as a ‘process’ emphasizes the act of receiving and interpreting feedback as a development process where feedback is part of a much larger, socially situated learning dynamic (Nicol 2010; Sadler 2010). In this way, feedback is acquired and exchanged through an ongoing dialogue and is not a possession of one person gifted to another (Wegner and Nückles 2015). This process view of feedback provision, more easily visualized as the opportunity for applicants to respond to reviewer comments prior to the decision being made, or else an interview stage, is not a model of peer review adopted by all funding agencies.

In peer review, there is a deficit between the level of assessment literacy between reviewers and applicants. Sustainable change to peer review as and developmental assessment system requires that both actors are aware of how to engage in feedback—how to send (reviewers), as well as receive and implement (applicants) commentary on unsuccessful grant applications in future career decisions including, but not restricted, to the option of reapplication of the proposal, strengthened by process feedback. In many occasions an applicant’s inability to manage their own equilibrium, or else underplay their own agency in actualizing their own improvement via feedback engagement (Winstone et al. 2017; Carless and Boud 2018), is also a major barrier in how feedback can contribute to future learning. This is especially the case if, and when, feedback is delivered alongside a strong and direct ‘reject’ signal. For this reason, the tone of communicating decisions, as well as the lens by which decisions are made, are vitally important to build a peer review system that doesn’t fail applicants, but instead is used to enable the type of process feedback that sends desirable ‘signals’ for future career development. Indeed, the persistent dissatisfaction in funding decisions made by peer review, demands a change in thinking about how we deliver decisions, as well as support future iterations and funding attempts.

## 3. Methods

### 3.1 Study design

This study was based on an analysis of successful and unsuccessful applications made to The Wellcome Trust, 2009–19 (*n* = 4105). Funding calls were chosen if they were targeted to ECR researchers and included the following funding calls: (1) Sir Henry Wellcome Postdoctoral Fellowship; (2) Sir Henry Dale Fellowship; (3) Research Career Development Fellowship; (4) Clinical Research Career Development Fellowship; and (5) Research Fellowships in Humanities and Social Sciences. Prior to 2021, applications for funding made to the Wellcome Trust were concentrated in two areas; (1) Clinical medicine; and (2) Medical humanities and social sciences.

**Figure 1.**
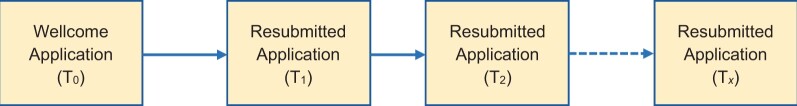
Pattern of resubmission underlying the study design.

The methodological approach in this study combined data from; (1) interviews with unsuccessful applicants from a survey, with (2) linguistic analysis of reviewer feedback. The conceptual pattern of resubmission following a unsuccessful outcome of an initial application to the Wellcome Trust (T_0_) that underpins this study is provided in Figure 1. The focus of the analysis in this study was on the transition between T_0_ (initial Wellcome Trust application) to T_1_ (reapplication to any funding organization) where feedback was received by applicants from The Wellcome Trust following an unsuccessful outcome at T_0_.

#### 3.1.1 *Interviews*

An initial survey (Derrick et al. 2021) of both successful and unsuccessful applicants to the Wellcome Trust (*n* = 233), identified a sample of unsuccessful applicants at T_0_ who were then invited to an interview (*n* = 19) to explore their career decisions and feedback received at T_0_.

During interviews, relevant excerpts from the feedback they received at T_0_ was presented to participants and used to stimulate discussion and used to explore participant reflections on how feedback motivated (explicitly and implicitly) personal and professional choices. No ‘ratings’ of the grant outcome were presented to the participants at this stage, as ratings are not part of this funding agency’s assessment. Instead, interviews explored how actual pieces of feedback were transmitted; received; understood and then implemented (Boud and Molloy 2013a,b) by applicants following T_0_. Interviews were conducted online via Zoom of Microsoft Teams, and were recorded and fully transcribed for analysis.

#### 3.1.2 *Linguistic analysis*

A random sample of *n* = 27 applications were analysed along with their corresponding reviewer comments (*n* = 81). Initial manual coding was cross-referenced with the literature on effective provision and implementation of feedback (Boud and Molloy 2013b; Ajjawi and Boud 2017; Carless and Boud 2018). Analysis was informed by previous linguistic approaches in analysing feedback and evaluation reports (Mann 2011; Ramani et al. 2019; Winstone and Carless 2019) including sensitivity to constructs of professional ‘politeness’ (Brown and Levinson 1987) and ‘face saving’ (Ginsburg et al. 2016, 2017).

Analysis of the interviews was inductive and completed in two inter-linked rounds: broad coding (memo-making and scanning interview transcripts for relevant themes) was performed independently by GD. Themes were discussed with the entire team which led to a more detailed, refined thematic coding (Braun and Clarke 2012) performed for a second time in line with the linguistic analysis.

### 3.2 Analysis: Combination of interview and linguistic analysis

A key characteristic of this study was the use of feedback received by an applicant at T_0_ as a tool during interviews with applicants who had received an unsuccessful outcome at T_0_. During interview analysis, the reactions of participants to particular characteristics of the feedback was monitored. In particular, any emotion responses as well as reflective narratives about how this feedback informed their next career decisions including, but not restricted to, the decision to reapply at T_1_. Analytic coding of interviews (GD) was initially conducted separately to the Linguistic analysis (AZ) with the latter developing the matrix shown above in Table 1.

**Table 1.** Triad of effective reviewer-comments-as-feedback based on the combination of interview outcomes and linguistic analysis

POSITIVE SIGNAL Characteristic associating with persisting	Definition	NEGATIVE SIGNAL Characteristic associated with switching
Actionable	*Comments perceived as feasible for the applicant to address or accomplish within 6-12 months and/or time for resubmission*	Unactionable
Targeted/specific	*Comments where there is no uncertainty as to what needs to be done; e.g. either identifying specific sections of the application to fix, providing specific references to read up on, or identifying new experiments.*	Vague
Fair	*Comments that are considered appropriate for the ECR career-level and/or within the personal capacity of the applicant*	Unfair

Outcomes from the initial rounds of coding from the interviews and peer reviewer reports were then compared, with the interview analysis further developing coding matrix for reviewer reports for the characteristics of feedback that were associated, by interview participants, as motivating their professional choices to ‘persist’ (reapply for funding to any funding organization at T1), or ‘switch’ (not reapply at T1; or else leave academia). This is shown in Table 1.

Where, in the interviews participants associated a particular feedback characteristic with an associated with behaviour motivation (switch or persist) in regards to their professional choices following a unsuccessful outcome at T_0_, then this was noted. This feedback characteristic was then given greater prominence in the feedback matrix shown in Table 1. This included feedback that applicants felt in control of addressing (Actionable), were relevant (Targeted), and that demonstrated appropriate quality thresholds for the ECR level (Fair).

The second round of coding of interviews took into account the participants reaction to feedback that contained one- or more characteristics, and participants association of the feedback as a whole (for survey results see Derrick et al. 2021). In summary, when the feedback received all three of these components (targeted, actionable and fair, Table 1), applicants considered the feedback as ‘good’ and ‘positive’. The absence all of these components were considered as ‘negative’ feedback. The results outlined below in the Results section centres on applicants’ descriptions and reactions to each feedback characteristic (Table 1) and outlines how this feedback was associated with an applicant’s decision to ‘switch’; or else ‘switch’ when the feedback was considered negative (unactionable, vague, unfair).

## 4. Results

### 4.1 Characterizing positive feedback in reviewer reports

#### 4.1.1 Targeted feedback

Targeted feedback was considered feedback that was directly relevant to the proposal and was not based on hypothesized assumptions of capability, ability or feasibility. Instead, targeted feedback was introduced by the reviewer in a tone that indicated the use of suitable expertise by using absent of uncertainty and assumption. This meant identifying specific sections of the applications to fix or change; identifying new experiments required prior to a resubmission; or else consideration of new arguments or literature to use in this resubmission. For example,

There has been some evidence that obesity is associated with variations in brain volume and in grey matter concentration or volume but these are not consistent. The PI might wish to consider whether an examination of brain grey matter differences is relevant to or essential for interpretation of the neuroimaging data: recognizing that attempts to control for grey matter or volume differences may introduce additional confounds or affect the interpretability of the data. **Reviewer comment (Targeted)**

Here, information about the additional variables to be considered by the applicant during resubmission that may ‘affect the interpretability of the data’, is offered in a tone that communicates suitable expertise and authority. In contrast, the use of words in the feedback below communicates uncertainty—such as ‘seems’ and ‘I think’ but did not always come as part of negative comments; ‘*I would describe the timeline as ambitious. However, I think it is achievable*’. In cases when doubt was associated with harmful feedback, reviewer use of qualifying statements to absolve themselves of excessively harsh commentary, or else use opinion to make claims they cannot support otherwise. However, in some cases there were direct admissions from the reviewer of their inability comment on certain aspects of the proposal, due to their lack of expertise.

In relation to scientific rigour, the candidate has failed to justify the use of (only) three cases studies. Is this sample scientifically significant? I will leave this question to social scientists and simply raise it here. **Reviewer comment (untargeted)**

For reviewers to diminutize their expertise, not only reflects negatively on the applicant but burdens the panel to navigate this uncertainty independently when making the final decision to fund, or not. For applicants, untargeted or vague comments are those without a direction of what needs to be fixed, such as *‘the methods are ill-defined’ or ‘there should be more detail in this section’*. Other untargeted or vague feedback included the reviewer’s insertion of themselves into the feedback. Here, the reviewer’s use of ‘I’, ‘me’ or ‘we’ in the feedback often led to the commentary associated with the reviewer’s *opinion* instead of independence from the proposal and the applicant. For example,

The various pathways led **me** to wonder whether or not the applicant was distracted or unfocused and ponder whether or not the form was untidy and poorly constructed because of lack of focus or other distractions. **(Vague)**

This self-insertion implied prestige signalling to an individual (example; *‘this journal doesn’t seem prestigious enough for me’* or ‘*I’m surprised they don’t do [suggestion], which I have found integral to my studies’*) and reflected a personal value or opinion that presented an unworkable (see Fair below) standard to the applicant.

#### 4.1.2 Actionable feedback

Actionable feedback envisioned a future for the applicant and application by providing constructive comments to be implemented. For the sake of the analysis, comments were only considered actionable if they could reasonably be completed within 12 months, allowing for enforcing necessary boundaries around reviewer comments.

Actionable feedback was targeted feedback in the presence of judgment-free steps aimed to encourage and increase the likelihood of success at resubmission. By doing so, actionable comments focused on what could be used to improve the proposal content and/or further articulating the ideas therein. The characterization of these comments echoed Carless and Boud (2018) focus on the provision of feedback that is useful and highlights opportunities for the recipient to take action while supporting greater assessment literacy. These comments were goal-related assessments of the proposal that were neutral and free from a judgement on where the proposal was going, and avenues for how to support to proposal to reach its goal. Actionable comments often recommended changes to methods or perspectives that, in the reviewers’ opinion, would improve the proposal and increase the likelihood of success at resubmission. For example, the below review provides two avenues for the applicant to consider in future iterations either change the title, or consider a shorter time focus;

I believe however that there is a problem with the design of the project and that the candidate should focus on a shorter period of time as the focus on *XXX* and his surroundings and the larger timeframe suggested in the title are inconsistent. **Reviewer comment (Actionable)**

In the above excerpt there is no judgement on which avenue the applicant should choose, but promotes the applicant’s learning by indicating proposal improvement scenarios (Hattie and Timperley 2007). However feedback is only effective if it is acted upon (Sutton 2012; Winstone et al. 2017) and some actionable comments are more easily adopted in future iterations than others. These included alternations such as adding references, new directions for thought, clarity of wording, or additional comments on the exclusion/inclusion of reviewer suggestions. Within this feedback there is a potential for self-interested bias to frame the interpretation of the feedback by the applicant, especially if it is assumed that suggestions from the literature stem not from a desire for the reviewer to improve future proposals. For example, in the next quote, the blinded nature of the peer review process means that it is difficult to interpret the motive for suggesting literature inclusions. It is therefore not clear how including this literature will improve the application;

Since a fellowship such as this offers the opportunity to work with the most appropriate groups to address a given problem. I would expect that the candidate should seek to bridge this gap by studying data from experimental epilepsy models and engaging with experts in this field……. other studies indicate diverse distributions for laminar states indicating different underlying mechanisms, see e.g. Suffczynski et al. (2006). **Reviewer comment (Actionable)**

In contrast to actionable comments, unconstructive feedback was also found. This included feedback that was considered without utility or comments that included unobtainable or impossible thresholds for improvement (e.g. suggests changing a fundamental aim of the project). Specifically unactionable comments were those that required a change but that would take more than a year to achieve. Examples here included a change in the host institution or supervisor; ‘*the lack of a senior scientist with expertise in functional imaging as a supervisor or collaborator is a concern*’; developing a stronger publication record; or else a major redesign of the project and therefore application. In these situations, these goals were technically possible but feedback was made without due consideration of the process of learning to achieve these goals. Unactionable feedback was also vague if it provided a judgement without a suggestion of the process of rectification for the applicant to consider ahead of any resubmission. For example,

‘With more care and attention to detail, especially regarding questions of access and method, this appealing anthropological collaboration could become a fundable proposition.’ **Reviewer comment (Unactionable)**

Here the goal was to become ‘fundable’ but in the absence of meaningful steps to achieve this goal.

#### 4.1.3 Unfair feedback

Not all unsuccessful applications are associated with unfair feedback. For the purpose of this research, unfair feedback was identified through a choice of words, or else commentary that was determined by the research team as to reasonably generate an emotional response by applicants. For example, the use of language indicating tentativeness right before the focal phrase, distorting an otherwise positive emotional impact, such as the following; *‘I assume the lack of detail was due to word limits of the project’ Reviewer comment (Unfair)*. Here, the comment could be interpreted as positive or negative depending on how the applicant would receive, interpret and then constructively implement this feedback (Sutton 2012; Carless and Boud 2018) but the illusions to *‘lack of detail’* suggest a reviewer motive that overlooks the emotional toll to applicants in receipt of the comment.

Relationships between reviewer comments that were coded as Unfair as well as Unactionable included suggestions for changes that were unworkable for an individual (e.g. change institutions, country or mentor) relative to their ECR status. These included insinuations of what a particular applicant was capable of at the ECR stage. As the below excerpt demonstrates, these comments included normative suggestions on what a ‘Fellow at this career stage’ should do in order to present and more fundable (ibid) application. The suggestion of being ‘*embedded in a group of imagers*’ or including more ‘*senior researchers*’, was a normative assumption of capabilities of the individual and therefore considered unfair;

However, there are no senior researchers in cognitive neuroscience involved in this application which is not ideal for a Fellow at this career stage who would be better placed being embedded in a group of imagers (even if they were working on different areas). **Reviewer feedback (Unfair)**

However, the majority of unfair comments were related to judgements of quality (ex-post) or potential (ex-ante) of the applicant based on thresholds that were not relevant to the ECR-stage. These included direct statements of a *‘poor track record’* that betrayed a misunderstanding of common restrictions placed on ECRs in pursuit of research independence and job security. For example, the comment; ‘*the most recent of these publications came out seven years ago. This is a poor track record, even taking into account her stage of career*’, does not consider why such a gap for this (female) researcher exists, but instead makes a judgement on a threshold of productivity above and beyond an appreciation for the individual circumstances that underpin such a gap. Other unfair comments within this theme of places unrealistic or unfair thresholds of research performance or excellence on ECR candidates, were considered unfair because they seemed to be applying thresholds of excellence that were more akin to an assessment of more established academics, and were ill-suited for ECRs, e.g. ‘*It is of some concern that he has not yet published a first author paper from his current lab*’. This also led to tensions between the reviewers’ sense of competitivity through performance, and the realities of an ECR establishing a level of career independence. Such comments overlooked the fact that the objective of ECR-followships was to provide the opportunity to develop research independence, and that having already established independence was not a fair pre-requisite for successful, and therefore fundable applications.

‘The candidate has a strong track record, but the degree of independence is unclear’ **Reviewer feedback (Unfair)**

In the below excerpt the reviewer is applying a performance threshold that is divorced from the reasonable performance expectations of applicants that are, as per the eligibility guidelines, up to 5 years post PhD. In addition, the reference to ‘top journals’ implies socially constructed terminology use that is embedded into the culture of which an ECR, by merit of being an ECR, is yet to become accustomed.

‘I have some reservations about the suitability of the candidate. For someone who has been working in the field with a PhD since 2009, the publication rate is extremely slow and the venues, with one exception, are not the top journals’. **Reviewer feedback (Unfair)**

Such comments suggest that for reviewers there is a tendency to apply to same performance standards and expectations on applicants with little consideration of their career status and developmental needs that are also relative to the career stage and/or the funding call objective.

### 4.2 Associating feedback received with professional decisions at T_0_

During interviews, participants were re-introduced to the feedback received for their unsuccessful application at T_0_. They were then asked to reflect how they interpreted the feedback, felt that it was relevant, and how it inspired and/or influenced their next career choices. The use of feedback as vignette’s allowed a more robust reflection of how feedback inspired proceeding career steps, with the (temporal) distance for these steps to be reflected upon free of the emotive reaction of any injustice perceived by applicants due to the unsuccessful application.

Interview analysis identified two applicant typologies based on the characteristics of participant’s career/research choices and likelihood that they would resubmit their T_0_ proposal in a similar form^1^ at T_1_. These were themed as ‘*Switchers*’ and ‘*Persisters*’.

*Persisters* were identified as applicants who reapplied at T_1_ after an unsuccessful submission at T_0_; whereas, *Switchers* were those who did not reapply at T_1_ or who had left academia after an unsuccessful submission at T_0_. How each typology responded to the characteristics of feedback described above received at T_0_ was explored in the interview analysis. In particular, how feedback sends ‘signals’ to applicants and, through these signals motivates behaviours for applicants to switch or persist was considered.

#### 4.2.1 Signals to persist

That participants were sensitive to signals received through reviewer-comments-as-feedback, compromises the consideration that competitive funding peer review is solely a process of selection. Participants reported how review comments affected their emotional state and, by extension, how they employed their own agency and feedback literacy (Winstone et al. 2017; Carless and Boud 2018). Participants also made assertions of reviewer motivations and temperament in receipt of the feedback as a meaning-making exercise. This was particularly the case when the feedback was negative. Applicants worked to personify the feedback given in a way as to reflect on the personal, and humanist motivations of the reviewer themselves;

So, I’m not sure whether the reviewer himself or herself knew the impact of their review; [that it means not], that it doesn’t mean that we are given the chance a chance to respond and improve the proposal, but it decides whether the proposal continues on or not. So in a way, yes the, the review is minimalist so probably in itself not negative, just asking for clarifications in defence, but in the context of the rejection ultimately, it feels like the negative review.

The sensitivity by which participants absorb these signals from feedback, and how these signals were internalized and then implemented in future research and career decisions, is an important reflection on the behaviour modification capability, and therefore the developmental potential of peer review. Participants primarily used these signals in feedback as a tool to decide whether to resubmit (persist) at T_1_, or not (switch).

The decision to persist (resubmit at T_1_) was motivated not only on the overall impression of positivity in the feedback where the majority of applicants felt that the reviewer knew them individually, or their work. It was also motivated by a positive orientation towards their own agency to implement to suggestions within the reviewer comments. This also acted to alter their perception that the peer review was conducted fairly, with appropriate reviewer expertise (targeted).

Yeah, there was one that was very positive, and I could also tell from the wording he or she knew me before because they made a comment that wouldn’t have made sense otherwise.

A signal to persist was embedded in how ‘useful’ the feedback was perceived towards resubmission at T_1_ (actionable), but also in how possible it was for the participant to act on that feedback. Since for ECRs, a resubmission at T_1_ is time-sensitive due to the eligibility of the applicant or else the novelty of the idea, feedback utility was related to how quickly and easily it would be incorporated into a renewed proposal. When feedback was used to stimulate learning, either about how to improve ‘grantmanship’ (McAlpine 2020) (‘*learn how to make applications*’) then this was seen as a signal by applicants to ‘persist’.

I let some time pass [after failure at T_0_] and then for my next applications I always reviewed the reviews. I always took into consideration what they said because it’s a process, one learns how to make applications

Despite a lack of success at T_0_, the applicants described how the feedback served not only as a tool to strengthen the application ready for resubmission (T_1_), but also is considered an important learning tool for the applicant at the ECR stage. For applicants who received feedback signals to persist, there was a sense that the feedback was considered useful, despite the negative outcome, if applicants retained a level of control over how it was to be implemented.

The feedback wasn’t particularly disturbing because I thought I can do something about the things which they appear to be most obviously criticizing.

If applicants felt in control of the suggestions made in the feedback, despite the decision not to fund the project, then this was interpreted as a signal to persist. In contrast, where this sense of control was lacking or else the feedback received did not stimulate learning was perceived as sending negative signals.

I didn’t feel it was useful. That’s for sure because it didn't teach me anything. It didn’t help me in any way to see flaws in the study design and improve the study for the future—so in terms of usefulness—No.

These signals were amplified by emotions caused by the strong signal sent by an unsuccessful outcome (failure), and hence did not encourage the applicant to persist. Further signals associated with the applicants’ decisions to switch are outlined in more detail below.

#### 4.2.2 Signals to switch

The decision of an applicant to ‘switch’ is not associated solely with the decision to leave academia or to never again apply for funding. The decision to leave academia, was considered an extreme form of switching, but was still a career choice exercised by a small number of interviewees (*n* = 3). More commonly, switching is associated with an applicant’s decision to change topic, discipline, to undergo retraining, or else to resubmit the T_0_ proposal in smaller, seemingly more competitive projects. The decision to switch, therefore, includes a range of behaviours and choices that result in either: the lack of an (re-application at T_1_); the lack of presence/visibility as an author in publications following the year of T_0_; and/or the presence of an application at T_1_ as a co-investigator only.

For applicants, receiving the news of an unsuccessful grant application was a strong signal to switch. This was amplified in situations where the news of a lack of success of an application was sent without any accompanying feedback. It should be noted here that for the Wellcome Trust, feedback is sent independently to the grant-decision. In the absence of reviewer comments, applicants felt frustrated not at the absence of feedback but by a sense of a lack of control that was explored in the previous section as a signal to persist; ‘*Yeah, it’s in a way, it doesn’t matter how negative each is in itself, when the outcome is that you don’t continue*.’*.* Applicants who did eventually receive feedback, reported that the absence of accompanying reviewers report at the time of receiving the funding outcome heightened the desire to switch. Indeed, applicants felt that at this time their ability to learn from the feedback, and therefore continue to improve their portfolio or the proposal, and therefore the prospect of persisting, was limited. The absence of feedback—positive or negative—meant that for the applicant the only signal received was the blunt ‘reject’ from the outcome, and therefore to switch; ‘*By that point you know that this means that you’re rejected*’. For participants, not all applicants received feedback and subsequently did not participate in the interview aspect of this research.

Feedback that solely communicates the rationale for the decision not to award funding, was disheartening and associated with a decision for applicants to switch. Such commentary was seen as devoid of relevance and as a tool of the funding agency and, by extension the reviewers, to promote authority irrespective of the characteristics of the applicant or their proposal. This reasserted an applicant’s perception of a lack of fit between themselves, their ideas and the potential for a future in the academy. In the excerpt below, as an example, the absence of feedback only acted to embed an impression of a lack of personal capability and sense of belonging;

I needed a while to see but they were saying, they were not saying, okay you are a fraud. They said ‘okay, we see that you have a background as a researcher and you can make some good stuff but this is not good enough for us and because this and that I’m sorry’ [you can’t be funded]

Other signals in the feedback associated with the decision to switch were more nuanced. As with the signals influencing a decision to persist, the level of perceived control an individual has over the future of the proposal, and its potential for eventual success is also important. As one participant explains, a successful project ‘*it can’t just be like an incremental research project, it needs to be transformatory (sic). It has to be transformatory (sic)*’. Feedback that was seen as lacking in envisioning a future for the proposal was also associated with a decision to switch. This was not related to simply the absence of ‘actionable’ feedback, but also feedback that contained actionable characteristics that were beyond the capabilities of the applicant to implement. Indeed, feedback provided in the absence of a consideration of whether it was within the capabilities, or even morally reasonable to assume an applicant would confirm in order to increase the likelihood of future success, also sent a signal to switch. For example, comments related to mobility and the perception that greater mobility would increase the competitivity of the proposal. These comments were actionable, but they were also unfair, and would signal the applicant to switch.

This is not reasonable to ask people to relocate, and no amount of family relocation costs can change people’s mental state when it comes to moving country.

Another common characteristic of actionable but unfair feedback was related to the assessment of the performance of ECRs at the stage of application. Feedback here concentrated on suggestions of how to increase performance but is also betrayed an expectation that an increased performance would result in a more competitive application both at T_1_. Above all else the type of feedback imposed unfair thresholds on ECR performance as well as unfair expectations of future academic performance.

So, you know someone said to me like… ‘they need to publish ten more articles’ then we’d have a problem because I honestly can’t publish ten more articles in the space of a year, you know at least not good ones.

Likewise, if feedback was not actionable in that it was unclear or confusing; ‘*It’s also very vague you know, it’s not easy to move it*’, then this was also interpreted by applicants as a decision to switch.

## 5. Discussion

This research shows a more nuanced vision for the role of peer review in the governance of academic research than has previously been considered in the literature. Though exploratory at this stage, the results indicate that reviewer-comments can work effectively as feedback to motivate potential persisters, and de-motivate switchers, when it contains the elements of being: actionable, targeted and fair. For ECRs at least, the interviews showed how the tone and utility of these reviewer reports have a strong influence in motivating their next career decisions, specifically as to whether ECRs with *persist* or *switch*.

This provides an opportunity to expand the understanding of peer review as it is currently conceptualized away from solely a selection tool, to one that can more holistically mobilize peer reviewer reports to act as feedback towards improving submissions and increasing the potential for success at T_1_ (participation/development). This re-imagining of peer review would also work to support applicants to persist in their academic careers and/or develop their research agenda even in the event of an unsuccessful outcome. For ECRs specifically, peer review plays an important tool in providing usable feedback from disciplinary peers that might not otherwise be received in order to strengthen research proposals and therefore the development of the individual. Considering that within the current peer review decision-making processes, the majority of applicants are unsuccessful, extending the function of peer review to include stronger developmental and participation elements could have wide ranging benefits to how research culture supports researcher generally especially for those at the early stages of their careers.

However, altering the mission of peer review implies adopting wide ranging changes for both funding agencies as well as for individual peer reviewers in how they approach the assessment of grant proposals. Indeed, the analysis of reviewer reports showed how peer reviewer comments as they are currently constructed are not always suitable to be provided to applicants as ‘feedback’ (Boud and Molloy 2013a,b) nor do they contain elements that are associated with applicant decisions to persist with a resubmitted proposal at T_1._ For applicants who persisted, reviewer comments that contained the elements of being targeted, actionable and fair, were associated with their decision to persist as well as success at T_1_. In contrast, feedback comments of the opposite characteristics (untargeted, unactionable and unfair), not only heightened the emotive reaction to feedback, but were also associated with an applicant’s decision to switch.

How the cognitive and emotional dynamics between applicants, applicants and reviewer-reports, complicates the peer review process (Cannon and Witherspoon 2005) and, more importantly, interrupts how applicants can interact with the feedback (Sutton 2012) towards a reapplication or else persisting in an academic career, is worthy of further analysis in studies of peer review. This study showed that, irrespective of the elements of feedback associated by the applicants as underpinning their decisions to persist (or switch), the emotive toll of either poorly constructed feedback cannot be overlooked. The approach here would be for funders to set clearer guidelines, or at least engage in essential reviewer training, about the standards and types of review comments expected prior to employing reviewers.

From the reviewers’ perspectives, wide ranging culture change is needed to ensure that reviewers are aware that their comments *is* used as feedback by applicants and is not solely, as is currently understood (Vallée-Tourangeau et al. 2022), as a binary selection mechanism (successful/unsuccessful). Instead, reviewer training should highlight that grant-peer review involves two objectives; (1) to assist the decision-making process (selection); and (2) to provide comments that can be utilized effectively by applicants as feedback (participation/development). This may require large scale changes to how researchers who act as peer reviews currently see their role of which previous research has seen as a series of dilemmas between self-interest (supporting familiar applications) and evaluation (rigorously assessing the proposal) (Lamont 2009; Vallée-Tourangeau et al. 2022). Whereas the dilemma surrounding self-interest driven reviews (Lamont 2009) may include a developmental consideration, there is still a lack of awareness of how good review commentary can act towards increasingly the likelihood of future success and, conversely the career and personal consequences of reviews that are ill-targeted or else, parsimonious.

The perspective adopted by this research is unique within meta-research in that it posits that efficient peer review processes is about more than making the right decision, reducing cost, or time, and instead highlights how greater and more invested participation by both reviewers and funding agencies can alter its governance role to one that includes a responsibility for the individual development of researchers and, potentially, as a tool to strengthen proposed research for funding overall. In this way, the research sees peer review as playing a role beyond ‘selection’, to one that focuses on ‘governance’ and ‘participation’ in research culture. In addition, in contrast to previous research of peer review and research careers, this research does not focus on events of success. Instead, a greater understanding of research selection and governance systems can be achieved through an understanding of the experiences of unsuccessful applicants, who are the majority and most probable outcome of current peer review processes. Indeed, from an equity and social justice angle, understandings experiences of failures can assist in repairing a system that is increasingly associated with bias (Lee et al. 2013), inefficiency (Roumbanis 2019), the risk of false-positive outcomes (Bornmann, Wallon and Ledin 2008; Lindner and Nakamura 2015; van den Besselaar and Sandström 2015), towards working better to serve the needs of ECRs and other vulnerable academics.

However, along with this approach, there are several limitations associated with this study including the low response rate. Whereas this low response rate may be the result of the timing of the survey (March 2020–July 2020) which corresponded with the initial lockdowns as a result of the COVID-19 pandemic, and the resulting disruptions. Another explanation might be that the along with a higher degree of mobility associated with ECRs comes the lack of access to old email accounts and the initial study invitation letter. In addition, whereas this article focuses on feedback, there are other factors including the ability of an institution to support an applicant to reapply that may influence an applicant’s decision to persist. Future studies should concentrate on gaining higher participation from ECRs, engaging a higher proportion of switchers as well as concentrate on how other research environmental factors complement peer review feedback in motivation ECRs to switch or persist. Future research may also use the conceptual model presented in this paper as a basis to examine predictors in peer review feedback in an applicant’s decision to ‘switch’ or ‘persist’.

## Footnotes

1 ‘a similar form’ relates to the understanding that a proposal at T_0_ is not always exactly the same proposal at T_1_. Applications at T_1_ can be: (1) the same; (2) a smaller pilot form of the project (less time, or less funding requested); or (3) in the same theme but still distinct in its methodological approach.
